# Low-Profile Antenna System for Cognitive Radio in IoST CubeSat Applications

**DOI:** 10.3390/s23104782

**Published:** 2023-05-16

**Authors:** Khaled Aljaloud, Kamel Sultan, Muhammad Ikram, Ali H. Alqahtani, Qammar Hussain Abbasi, Rifaqat Hussain

**Affiliations:** 1 College of Engineering, Muzahimiyah Branch, King Saud University, Riyadh 11451, Saudi Arabia; 2 School of Information Technology and Electrical Engineering, the University of Queensland, Brisbane, QLD 4072, Australia; 3 James Watt School of Engineering, University of Glasgow, Glasgow G12 8QQ, UK; 4 Independent Researcher, London E1 4NS, UK

**Keywords:** CubeSats, reconfigurable antenna, cognitive radio, internet of space things, UHF band

## Abstract

Since the CubeSats have become inherently used for the Internet of space things (IoST) applications, the limited spectral band at the ultra-high frequency (UHF) and very high frequency should be efficiently utilized to be sufficient for different applications of CubeSats. Therefore, cognitive radio (CR) has been used as an enabling technology for efficient, dynamic, and flexible spectrum utilization. So, this paper proposes a low-profile antenna for cognitive radio in IoST CubeSat applications at the UHF band. The proposed antenna comprises a circularly polarized wideband (WB) semi-hexagonal slot and two narrowband (NB) frequency reconfigurable loop slots integrated into a single-layer substrate. The semi-hexagonal-shaped slot antenna is excited by two orthogonal +/−45° tapered feed lines and loaded by a capacitor in order to achieve left/right-handed circular polarization in wide bandwidth from 0.57 GHz to 0.95 GHz. In addition, two NB frequency reconfigurable slot loop-based antennas are tuned over a wide frequency band from 0.6 GHz to 1.05 GH. The antenna tuning is achieved based on a varactor diode integrated into the slot loop antenna. The two NB antennas are designed as meander loops to miniaturize the physical length and point in different directions to achieve pattern diversity. The antenna design is fabricated on FR-4 substrate, and measured results have verified the simulated results.

## 1. Introduction

With the advanced varieties of wireless applications along with addressing the requirements of end user demands which can be varied daily, especially during disaster situations, a flexible communication system is necessary. Numerous use cases require a more global, scalable, flexible, and robust solution, such as monitoring remote areas, internet provisioning to underserved or disturbed regions, or intelligent global transport management. CubeSats are recommended to achieve global communication systems between those different services [1,2,3]. CubeSats are a type of low-weight satellite (1.33 kg) that have a volume of 10×10×10 ${\mathrm{cm}}^{3}$ and a weight of less than 10 kg (commonly represented as 1 U). Their small weight considerably lowers launch costs, making the idea of multiple satellites constellation in orbit possible. Several CubeSats have been proposed in the last couple of years, such as the RainCube precipitation radar, Internet of Space Things (IoST), space exploration, rural communication, remote sensing, and other contemporary CubeSat constellation projects [1,4,5,6,7,8,9,10,11].

**Figure 1:**
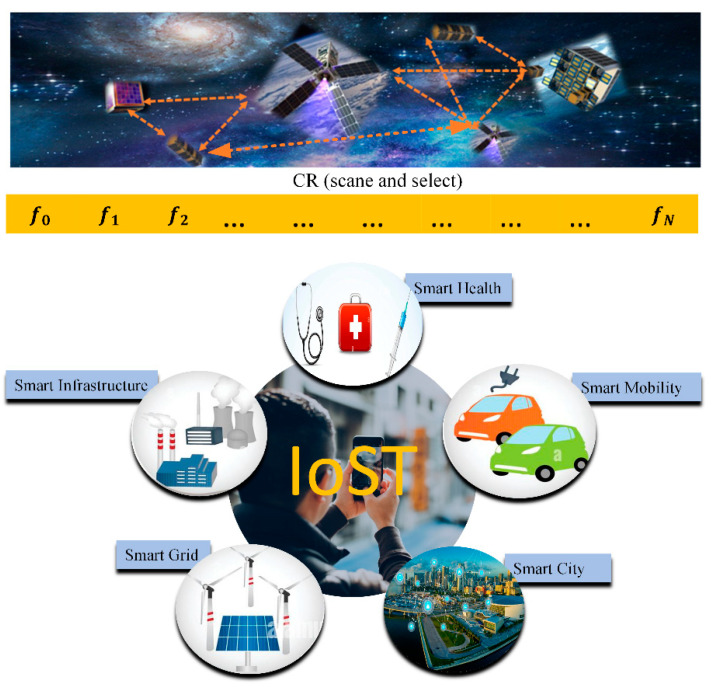
The conceptual scenario of satellite services.

In order to solve the connection problems between multiple CubeSats, the cognitive radio (CR) offers a potential solution. In CR communication, a transceiver is able to discern between channels that are being used and those that are not. It avoids occupied channels and rapidly enters vacated ones without interference with the licensed user. Thus, CRs are a class of intelligent transceivers with increased situational awareness due to their cognitive skills. This can result in the improvement of the efficient and robust use of communication resources and low delay of data exchange in the CubeSat constellation [5,9,12,13] (see Figure 1). While to solve the problems of globality services, the IoST, a pervasive cyber-physical system that will enable full global communication, is recommended (see the IoST services in Figure 1). The use of CubeSat does not limit to previous applications. Still, it extends to serve low data rate links for telecommand, telemetry, and control data for 5G networks at very high frequency and ultra-high frequency (VHF/UHF) [14,15,16].

Due to space restrictions, achieving multifunctional antennas in the CubeSat is challenging. Indeed, the antenna is the main key for the CubeSat to provide those aforementioned services (CR and IoST); so, a wideband antenna for sensing and a reconfigurable narrowband antenna for communication in low profile and compact size is mandatory. Thus, some physically changing antenna techniques such as origami folding, hinges, soft robotics, spring forces, and telescopic actuation are implemented [17,18,19,20,21,22,23], but those methods significantly increase the mechanical complexity of the CubeSat.

Extensive work has been undertaken to provide antennas for cognitive radio applications [24,25,26,27,28,29,30,31,32,33], but most of them do not fit with CubeSat regarding operating frequency, size, and compatibility. Although several designs have been employed for the CubeSat [34], such as slot antennas [8,35], the spiral antenna [36], helix [37], aperture antennas [38,39], superstrate antenna [40], solar cell-integrated antennas [40,41,42,43], dipole antenna [44], and folded dipole antenna [45], they do not support the UHF band with compact size and simple structure.

In this paper, a simple structure-folded slot antenna design has been presented. The proposed antenna consists of three antennas; one works as wideband antenna which is fed by two feedlines to provide circular polarization, while the other two are narrowband antennas that are fed by separate feedlines in a different orientation, to achieve radiation diversity. In addition, each NB antenna has a varactor diode. The NB antennas change their frequency over the operating band of the wideband antenna. By changing the value of the loading capacitor, the antenna will achieve frequency reconfigurability. The proposed antenna with reconfigurability features in addition to wideband at UHF can be a good candidate for the Cubesate of IoST applications.

## 2. Antenna System Structure

The proposed antenna is designed on a 100 mm×100 mm FR-4 substrate with a dielectric constant of 4.4, a loss tangent of 0.02, and a thickness of 1.52 mm. The proposed antenna system integrates a circularly polarized wideband antenna for sensing and two reconfigurable narrowband antennas for communication.

### 2.1. Wideband Antenna

**Figure 2:**
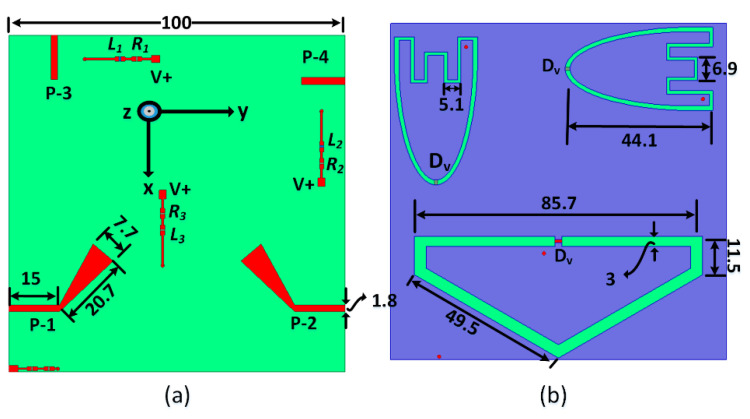
The antenna system structure (all dimensions in mm). (**a**) Top view and (**b**) bottom view.

The sensing antenna is a semi-hexagonal-shaped slot antenna with a perimeter $\approx of\;0.85\;\lambda_{g}$ at the center of the required band (0.7 GHz). The loaded capacitor at the middle of the slot’s baseline with a value of 0.38 pF (see Figure 2) results in the slot’s small physical length and improves the impedance-matching bandwidth. The width of the slot is 3 mm.

**Figure 3:**
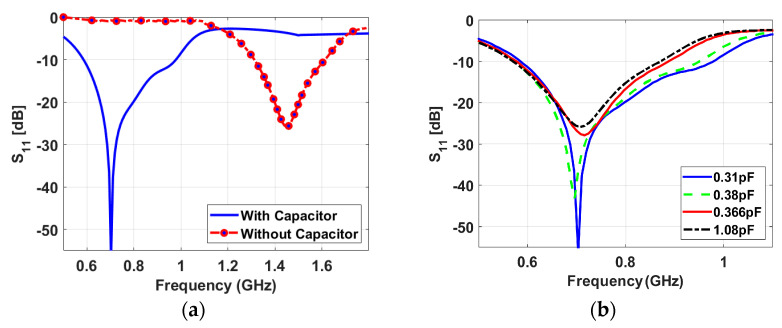
S-parameters of the sensing antenna (**a**) with and without capacitive loading, (**b**) for various capacitive loadings.

**Figure 4:**
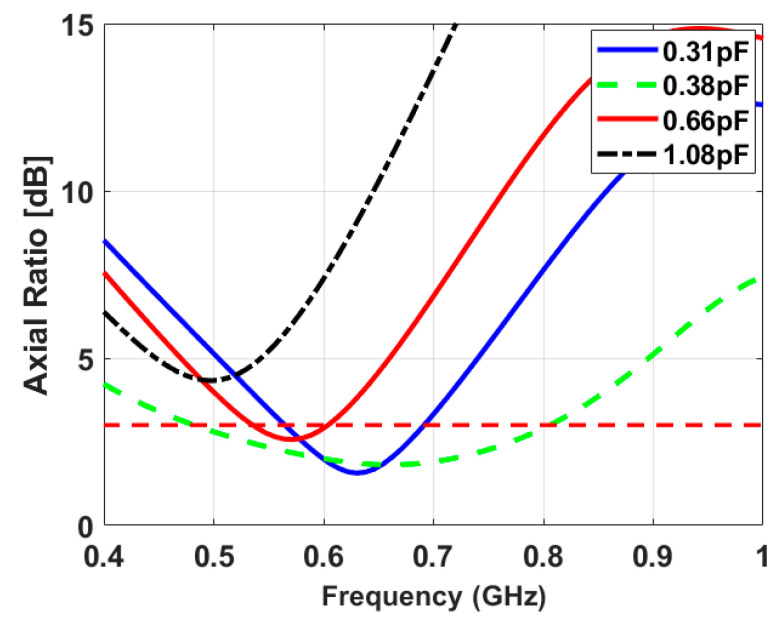
Axial ratio curves for various capacitance values.

Figure 3 shows that the sensing antenna operates from 1.3 GHz to 1.62 GHz before loading the capacitor, while it is reduced to cover a band from 0.57 GHz to 0.95 GHz in case of loading the capacitor. The slot is fed by two tapered transmission lines, the left feed (P-1) excites LHCP, and the right feed (P-2) excites RHCP. The axial ratio of the proposed antenna has been presented versus frequency at different capacitor values in Figure 4. The axial ratio with a capacitor value of 0.38 pF confirms the circular polarization of the antenna.

**Figure 5:**
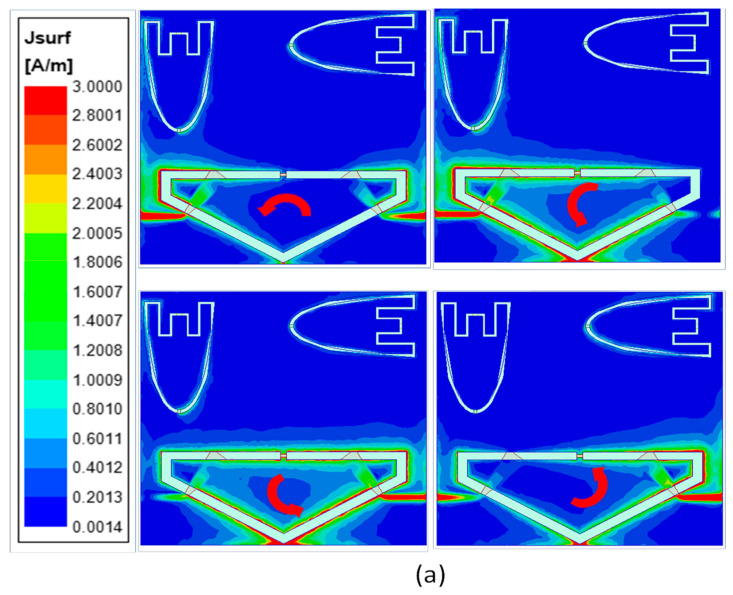

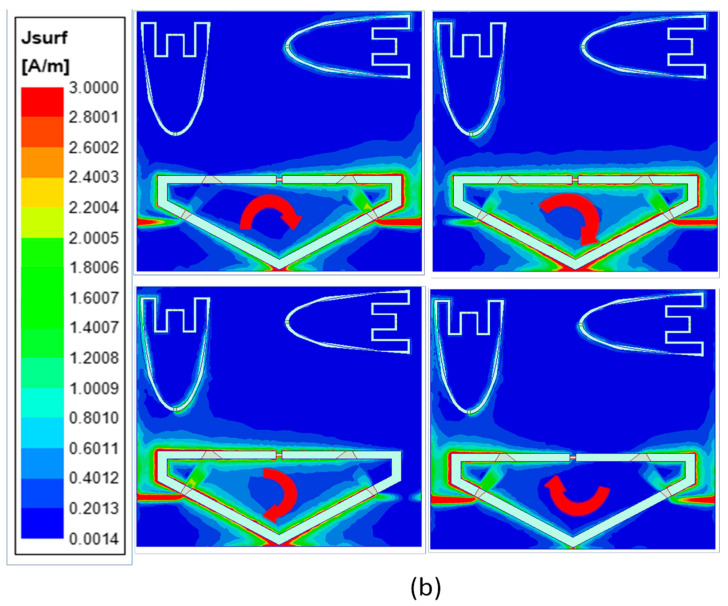
Current distribution for phases at 0°, 45°, 135°, and 180°, when the antenna is excited from (**a**) P-1 and (**b**) P-2.

To better understand the CP generation in the left- and right-hand sense, the surface current distributions are provided in Figure 5. When P-1 is excited, the current distributions are plotted at angles of 0°, 45°, 90°, and 180° at 0.6 GHz using High-Frequency Structured Simulator (HFSS), as depicted in Figure 5a. It is observed that the current distribution varies in the clockwise direction, which produces left-handed circular polarization. Similarly, when the antenna is excited from P-2, the current directions vary in the counterclockwise direction (see Figure 5b), demonstrating the antenna’s ability to radiate as right-handed circular polarization.

### 2.2. Narrowband Reconfigurable Antenna

**Figure 6:**
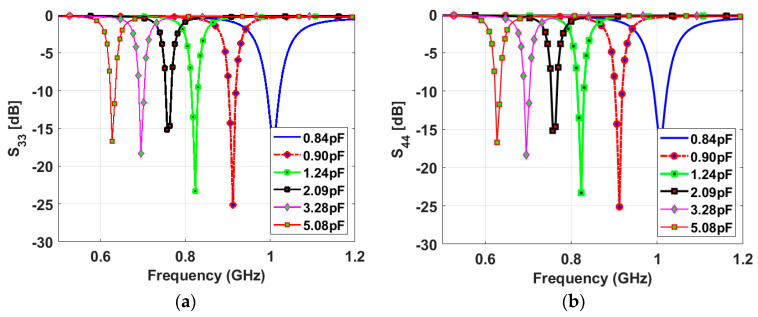
Simulated reflection coefficients of the NB antennas with different values of the capacitor: (**a**) *S_33_* and (**b**) *S_44_*.

**Figure 7:**
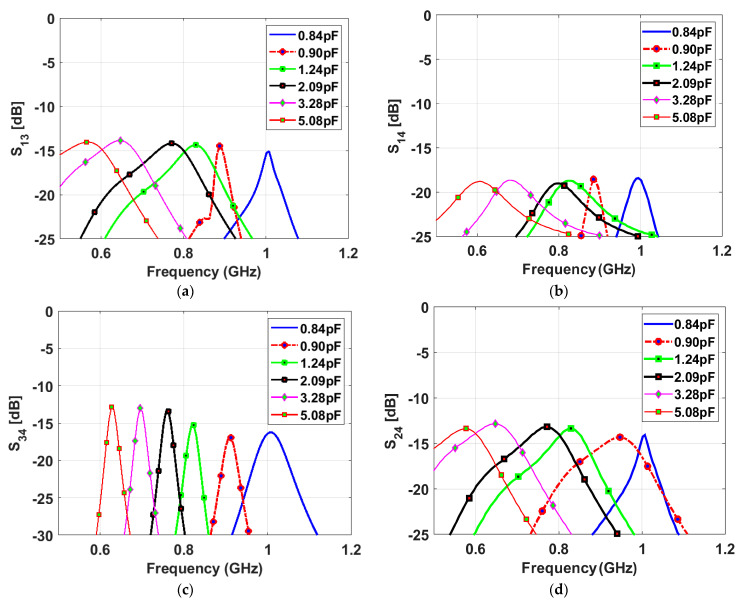
Simulated isolation coefficients between the NB antennas and WB antenna with different values of the capacitor (**a**) between P-1 and P-3 (between WB and NB), (**b**) between P-1 and P-4 (between WB and NB), and (**c**) between P-3 and P-4 (between NB and NB) and (**d**) between P-2 and P-4 (between WB and NB).

The narrowband antenna is a semi-ellipse-meander-shaped slot antenna; the meander shape is employed to reduce the antenna’s physical size. Similar to the sensing antenna, the NB antenna is loaded with various capacitance values from 0.84 pF to 5.08 pF. A varactor diode is connected to the antenna to reconfigure its operating frequency based on the bias voltage value, which changes the capacitor value. The proposed slot antenna has a total length of 86 mm and a width of 32 mm. Two NB antennas are printed on the same view with the sensing antenna. They are printed in two different directions to achieve pattern diversity. Five capacitance values (0.84, 0.9, 1.24, 2.09, and 5.08 pF) are used to tune the antenna’s resonant frequency over the wide frequency band. Figure 6 depicts the reflection coefficients of different capacitance values for NB P-3 and NB P-4. The results show a good match in all cases, and the resonant frequency decreases with increasing the capacitance value. High isolations between the NB P-3/NB P-4, NB P-3/WB-P1, and NB P-4/WB-P1 have been achieved thanks to the different orientations of the antennas (see Figure 7).

The biasing circuitry of the reconfigurable antenna, as shown in Figure 2, consisted of the varactor diode, RF choke (L_1_, L_2_ = 1 μH), and current-limiting resistor (R_1_, R_2_ = 2.1 KΩ). The varactor diode (SMV 1233) is connected to the biasing circuitry through shorting posts. RF chokes are used to separate the radiating structure from the DC power source while the reverse-biased varactor diode serves as a DC blocking capacitor to ensure that the DC biasing component and the RF radiating structure are well isolated.

## 3. Antenna Fabrication and Discussion of Results

**Figure 8:**
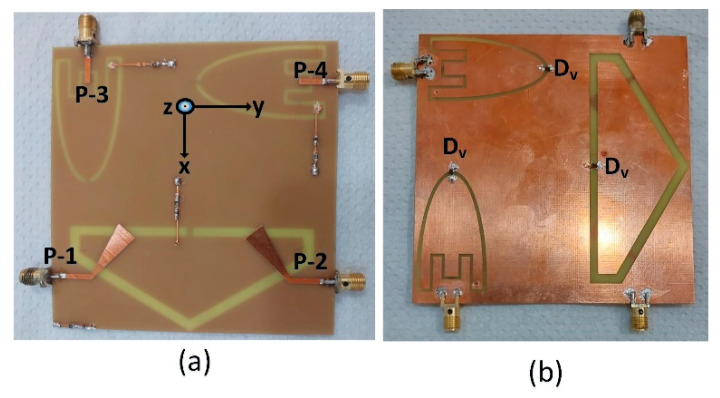
Photograph of the prototype of the proposed antenna: (**a**) front view and (**b**) bottom view.

Figure 8 illustrates the fabricated prototype of the proposed antenna system with four ports. The fabrication was done using chemical etching which is a popular method to remove excess copper from the surface of a copper-coated substrate. This technique involves coating the copper surface with a photosensitive material called “resist”, exposing it to light through a mask to create a circuit pattern, and then developing the resist to create an image of the circuit pattern.

Next, the board is placed in an etchant solution that selectively dissolves the unprotected copper areas, leaving behind the desired circuit pattern. The etchant solution usually contains chemicals such as ferric chloride, ammonium persulfate, or cupric chloride, which react with copper to dissolve it. Once the required amount of copper has been removed, the board is rinsed with water to eliminate any residual etchant solution, and the resist is removed, revealing the final circuit pattern on the copper-coated substrate. Chemical etching is a highly reliable and widely used method that enables the production of high-quality PCBs with precise circuit features. The proposed antenna was measured for its S-parameters and radiation characteristics.

### 3.1. Scattering Parameters

**Figure 9:**
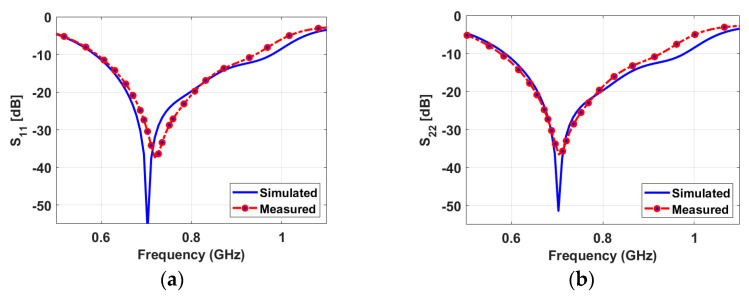
Simulated and measured reflection coefficients of the wideband (sensing) antenna: (**a**) at port-1 and (**b**) at port-2.

**Figure 10:**
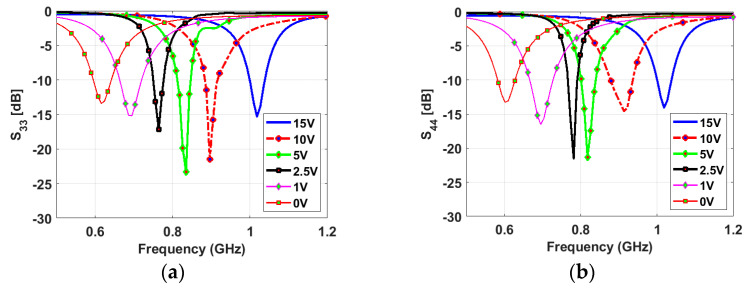
Measured reflection coefficients of the NB antennas: (**a**) NB-P3, (**b**) NB-P4.

**Figure 11:**
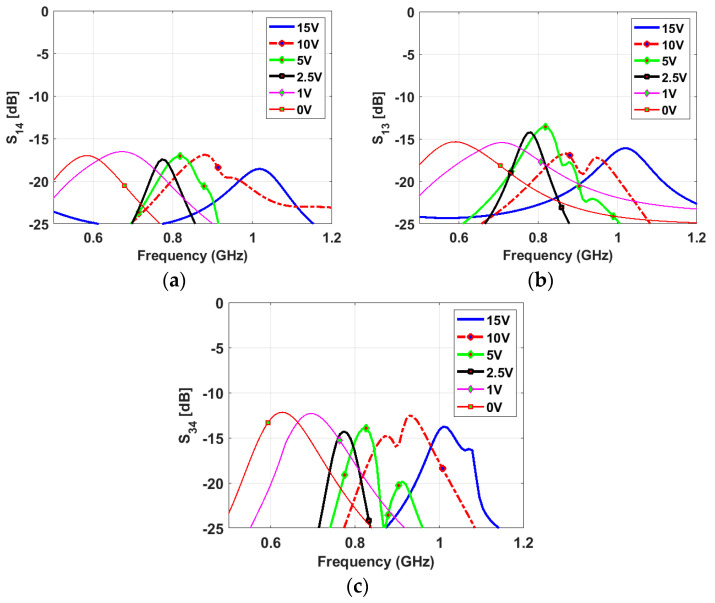
Measured isolation coefficients: (**a**) between NB-P3 and WB-P1, (**b**) between NB-P4 and WB-P1, and (**c**) between NB-P3 and WB-P1.

Figure 9 shows a good agreement between the simulated and measured reflection coefficients of the wideband antenna. The results confirm that the antenna operates from 0.59 GHz to 0.95 GHz numerically and experimentally with a good matching over the wide band. Figure 10 presents the measured reflection coefficients of narrowband antennas at the corresponding voltages of the above-mentioned capacitors values. The bias voltages of 15, 10, 5, 2.5, 1, and 0 V are applied consecutively to the varactor diode for both NB antennas, resulting in the resonant frequency’s reconfigurability from 0.6 GHz to 1 GHz. All the reconfigurable cases have good matching. In addition, high isolations between the WB antenna and NB antennas have been validated in Figure 11a,b, with an isolation factor of more than 15 dB in all cases. The high isolation between NB-P3 and NB-P4 has been validated in Figure 11c.

### 3.2. Radiation Characteristics

**Figure 12:**
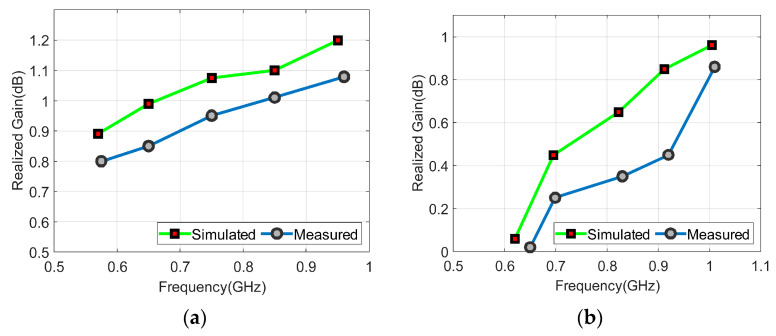
Peak gain curves: (**a**) Sensing antenna, (**b**) frequency reconfigurable antenna.

**Figure 13:**
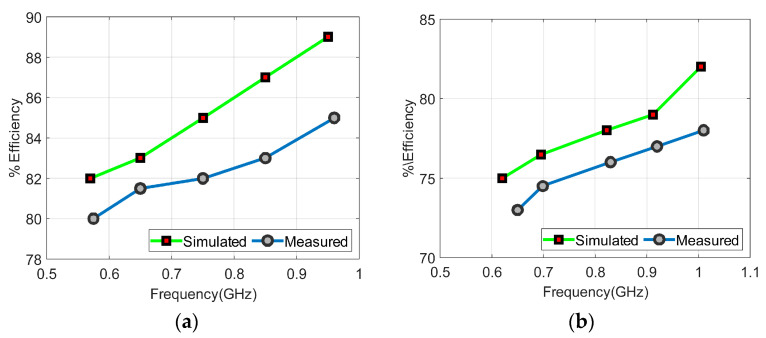
Radiation efficiency: (**a**) Sensing antenna, (**b**) frequency reconfigurable antenna.

The far-field radiation pattern characteristics were also performed for the proposed MIMO antenna. To calculate the antenna’s performance, the peak gain and radiation efficiency values (%η) are determined for each element of the antenna. To conduct the measurement for any port, the rest of the MIMO antenna ports were terminated with a 50 Ω load to avoid any reflected power. For sensing and frequency reconfigurable antennas, the peak gain values were 1.079 dBi and 0.86 dBi, respectively, while %η varied from 80~85% to 73~78%, respectively. Figure 12 and Figure 13 show the simulated and measured peak gain and %η curves for both sensing and frequency reconfigurable NB antennas.

**Figure 14:**
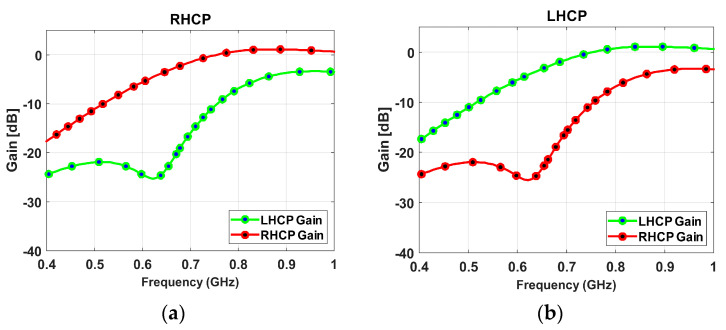
Peak gain curves: (**a**) RHCP, (**b**) LHCP.

Figure 14 shows the plots for the antenna gain of port-1 and port-2 in terms of left-hand circular polarization (LHCP) and right-hand circular polarization (RHCP). In port-1, the antenna’s RHCP gain is higher than its LHCP gain, and it can reach a maximum value of 1.079 decibels relative to an isotropic radiator (dBi) within the antenna’s operational frequency band. Similarly, for port-2, the LHCP gain is greater than the RHCP gain, with a maximum value of 1.078 dBi achieved.

**Figure 15:**
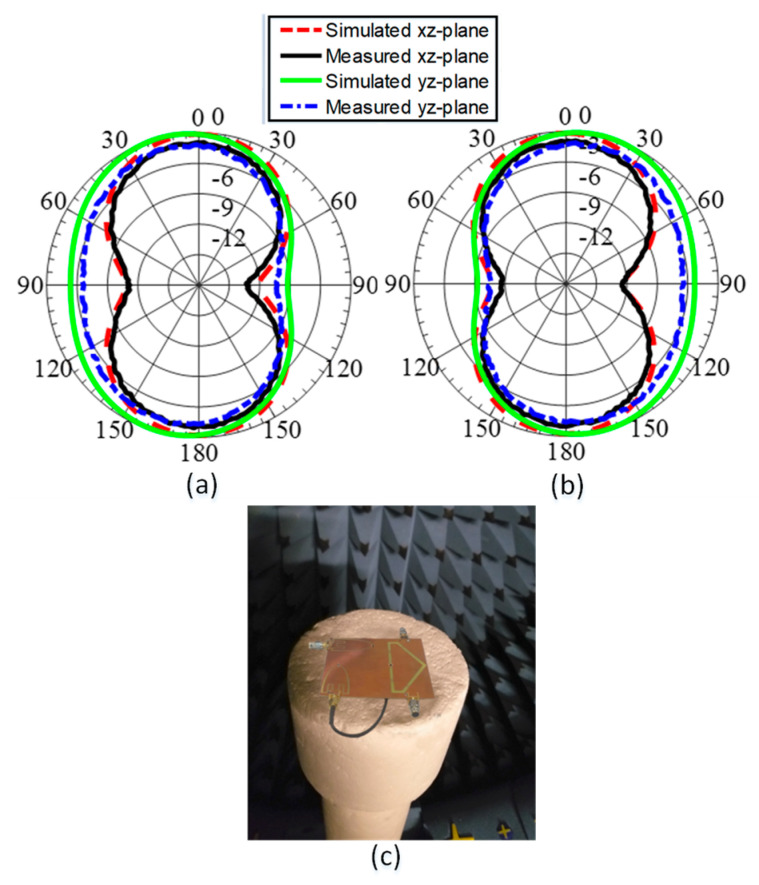
Simulated and measured radiation patterns of the wideband antenna in xz- and yz-plane. (**a**) P-1 and (**b**) P-2. (**c**) Measurement setup.

**Figure 16:**
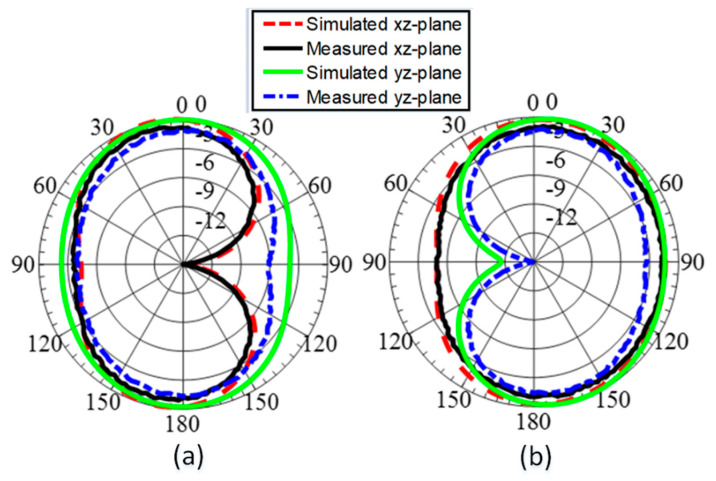
Simulated and measured radiation patterns of the narrowband antenna in xz- and yz-plane. (**a**) P-3 and (**b**) P-4.

The measured and simulated radiation patterns for the UWB antenna and narrowband reconfigurable antenna are shown in Figure 15 and Figure 16, respectively. The far-field measurement setup is shown in Figure 16c. The proposed antenna was characterized by its far-field measurements in an anechoic chamber. An anechoic far-field measurement setup typically includes the following components:

1. Anechoic chamber: The chamber is large enough to accommodate the antenna under test and other required testing equipment. The walls, ceiling, and floor of the chamber are covered with radiation-absorbing material to prevent reflections and create an environment free of external interference.
2. Antenna positioning system: The antenna positioning system is used to precisely control the position and orientation of the antenna under test. This system typically includes a rotation stage and one or more translation stages, which allow the antenna to be positioned at various distances and angles from the measurement equipment.
3. Signal generator: The signal generator is used to generate the RF signal that excites the antenna under test. The frequency and power level of the signal can be adjusted as needed.
4. RF receiver: The RF receiver is used to measure the signal received by the antenna under test. The receiver is typically connected to an antenna or probe that is positioned at a fixed distance and angle from the antenna under test.
5. Data acquisition system: The data acquisition system is used to collect and store the measurement data. This system includes a computer, software, and any necessary interfaces for controlling the measurement equipment and recording the data.
6. Calibration equipment: The calibration equipment is used to calibrate the measurement system and ensure accurate results.

The patterns show omnidirectional radiation patterns in both planes for wideband antenna, indicating they are useful for UHF band communication applications. The NB antennas have omnidirectional radiation and wide-beam directive patterns, alternating in both planes which makes them useful for sensing behavior. The simulated and measured results show good agreement for both antennas in both XZ and YZ planes.

### 3.3. MIMO Diversity (ECC)

**Figure 17:**
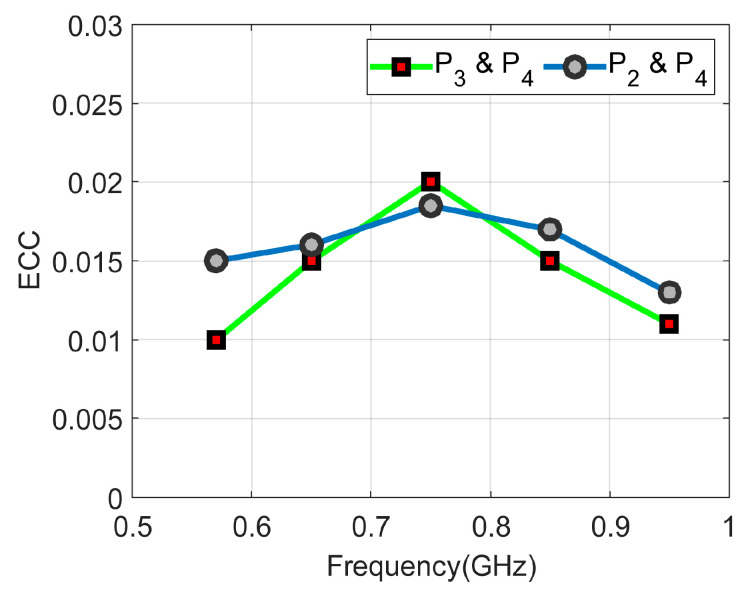
ECC curves between various antenna elements.

The diversity of the MIMO antenna in terms of envelope correlation coefficient (*ECC*) is calculated to show how much antenna elements are independent in their performance. The values are found to be very low, less than 0.02, ideal for the MIMO operation. ECC values are calculated based on the radiation patterns of antenna elements as given below:

(1) $$ECC_{ij}=\frac{{\left|\iint_{0}^{4\pi }\left[\vec{E_{i}}\left(\theta ,\;\varphi \right)\times \vec{E_{j}}\left(\theta ,\;\varphi \right)\;\right]d\Omega \right|}^{2}}{\iint_{0}^{4\pi }{\left|\vec{E_{i}}\left(\theta ,\;\varphi \right)\right|}^{2}d\Omega \iint_{0}^{4\pi }{\left|\vec{E_{j}}\left(\theta ,\;\varphi \right)\right|}^{2}d\Omega \;}$$

where $\vec{E_{i}}\left(\theta ,\;\varphi \right)$ is the radiation pattern of the *i*th antenna element. Figure 17 shows the ECC curves for both sensing and frequency reconfigurable antenna elements. It is evident that both antenna elements are performing well over the desired bands of operation.

### 3.4. Comparison

The proposed antenna features the benefits of sensing and communication integrated antenna, compatibility with unity CubeSat size, low profile, dual CP sensing, communication diversity, frequency reconfigurability, and UHF support. Table 1 lists a comparison between the proposed antenna and the referenced CubeSat antennas. It is easy to notice that pattern diversity has been achieved in [35,44] based on complicated 3D structures. The dual polarization has been achieved in [8] as CP and in [46] as LP without pattern diversity and reconfigurability based on slot antenna and shared aperture antenna, respectively. On the other hand, the antennas in [3,8,38,40,47,48] achieved CP with a complicated structure. Therefore, the proposed antenna is a good candidate for future CR-IOST applications.

## 4. Conclusions

This work presents a low-profile WB and NB frequency reconfigurable antenna for IoST applications. The proposed antenna consists of a circularly polarized WB semi-hexagonal slot and two NB frequency reconfigurable loop slots integrated on the same substrate board. The semi-hexagonal-shaped slot antenna is excited by two orthogonal ±45^o^ tapered feed lines and loaded by a capacitor in order to achieve left/right-handed circular polarization in a wide bandwidth from 0.57 GHz to 0.95 GHz. The frequency reconfigurable antenna operates over a wide band from 0.6 GHz to 1.05 GH using a single varactor diode per antenna element. The two NB antennas are designed as meander loops to miniaturize the physical length and reactive loading, which further optimized it to be operated in sub-GHz bands. The antenna system is fabricated on FR-4 substrate with a dimension of 100 × 100 × 0.76 mm^3^. The measured results verified the simulated results and both are in good agreement. The proposed antenna design is well suited for IoST CubeSat applications.

## Figures and Tables

**Table 1:** Comparison between reported antennas and the proposed antenna.

Ref.	$\mathrm{Size}\;\left(mm^{3}\right)$	f (GHz)	Reconfigurability	Low Profile	Support UHF	Pattern Diversity	Polarization	Remarks
[3]	87×87×27.5	1.575	No	No	No	No	CP	Helix
[8]	60×60×1.52	0.4	No	Yes	Yes	No	Dual CP	Slot antenna
[35]	100×100×100	0.45	No	No	Yes	Yes	LP	Folded slot antenna
[38]	100×100×25	0.9/5.8	No	No	No	No	CP	Microstrip antenna with shaped ground + Fabry Perot
[40]	100×100×7.2	2.5	No	Yes	No	No	CP	2 Substrate + 2 foam layers
[44]	100×100×100	6	Yes	No	No	Yes	LP	3D structure
[47]	110×110×3.18	1.575/2.2	No	Yes	No	No	CP	Stacked patch antenna of three layers
[46]	100×100×1.6	3.2/9.3	No	Yes	No	No	Dual LP	Shared aperture antenna
[48]	100×100×5.5	1.69/2.45	No	Yes	No	No	CP	Conical shape spiral antenna
[49]	NA (1.5 U)	0.48	No	Yes	Yes	No	LP	Slot antenna on solar panel
[50]	159×152×3.25	2.4	Manual	Yes	No	No	LP	Patch/monopole antenna
Proposed	100×100×1.52	0.55	Yes	Yes	Yes	Yes	Dual CP	Sensing antenna + two communication antennas

LP: linear polarization. CP: circular polarization.

## Data Availability

Not applicable.
